# Case report: special imaging features in a uremic patient with intracranial infection caused by *Ralstonia mannitolilytica*, almost misdiagnosed as demyelinating disease

**DOI:** 10.3389/fmed.2024.1513771

**Published:** 2025-01-14

**Authors:** Hanxin Kong, Hao Ying, Jianhong Yang

**Affiliations:** Department of Neurology, Ningbo First Hospital, Ningbo, Zhejiang, China

**Keywords:** *Ralstonia mannitolilytica*, next generation sequencing (NGS), intracranial infection, uremic, peritoneal dialysis

## Abstract

*Ralstonia mannitolilytica* is a very rare pathogen that causes intracranial infection. It is commonly found in immunocompromised patients and is resistant to multiple antibiotics. In this case report, we present a case of human central nervous system infection caused by *Ralstonia mannitolilytica*, which was initially misdiagnosed as demyelinating disease due to the specific imaging findings. This case had concurrent uremia and a good response to meropenem under continuous peritoneal dialysis. The diagnosis is mainly based on cerebrospinal fluid analysis and targeted genetic testing by next generation sequencing (NGS). However, the patient had a poor prognosis due to uncontrollable gastrointestinal bleeding and related complications during long-term bed rest. We hope that this case will attract more attention and provide relevant reference for the diagnosis and treatment of other similar patients.

## Introduction

*Ralstonia mannitolilytica* is an obligate aerobic Gram-negative bacterium widely distributed in nature. It is a rare opportunistic pathogen of nosocomial infection, especially in patients with cystic fibrosis and organ transplantation, which can cause bacteremia (1–4), meningitis (4), sepsis (5), peritonitis (6), osteomyelitis (7), hemoperitoneum (7), urinary tract infection (8), etc. Outbreaks of *Ralstonia mannitolilytica* infection in pediatric patients across the United States have been reported due to contamination of oxygen equipment (4, 8). Encephalitis caused by this bacterium is very rare. This is the first report of *Ralstonia mannitolilytica* encephalitis in a uremic patient who was almost misdiagnosed as demyelinating disease.

## Case presentation

**FIGURE 1:**
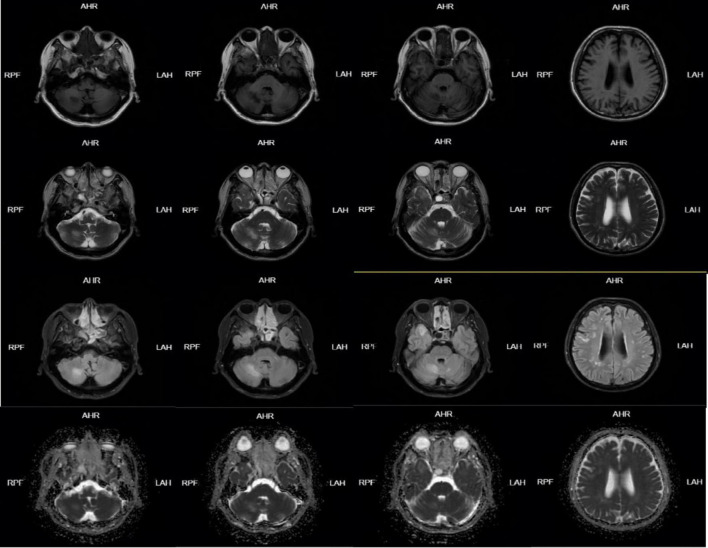
Head MRI on January 05, 2024-01 showed abnormal signal lesions in bilateral fronto-parietal subcortical cortex, right temporal lobe and right cerebellar hemisphere. Patchy long T1 and long T2 signal shadows were found in the right temporal lobe and right cerebellar hemisphere, FLAIR showed high signal, DWI and ADC showed equal signal, and scattered patchy long T1 and long T2 signal signals were found in the bilateral fronto-parietal subcortical cortex. FLAIR showed hyperintensity, DWI and ADC showed isointensity.

**FIGURE 2:**
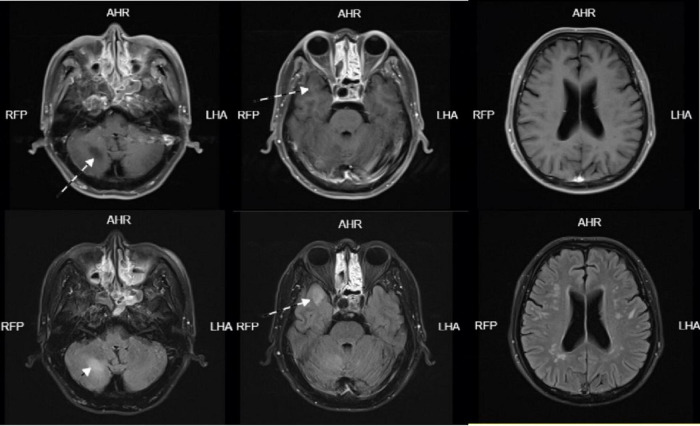
Head enhanced MRI on January 07, 2024-01: Scattered speckle-like slightly long T1 and slightly long T2 signal in bilateral frontoparietal and periventricular white matter, FLAIR sequence showed high signal, DWI showed no obvious high signal, no obvious enhancement. Patchy long T1 and long T2 signals were seen in the right temporal lobe and right cerebellar hemisphere, which were hyperintense on FLAIR sequence. DWI showed no obvious hyperintense signal and no obvious enhancement. A circular long T1-long T2 signal was seen in the right temporal base, with clear borders, measuring approximately 18 × 12 mm, and no enhancement.

On December 5, 2023, a 57-year-old male patient with uremia developed persistent dizziness, drinking cough, and dysphagia without obvious triggers. The patient went to the local hospital for head CT examination, and no obvious abnormal signs were found. Blood test showed high sensitivity C-reactive protein: 5.56 mg/L, white blood cell count: 6.8 × 10^9/L. The patient denied that there was a history of infection before the illness, and there was no fever at this time, so no attention and further diagnosis and treatment were given. On January 5, 2024, the patient developed slurred speech and weakness of the right upper limb, which was characterized by persistent fine activities. The head magnetic resonance imaging (MRI) was performed again at the local hospital, which showed abnormal signal lesions in the bilateral frontoparietal subcortical cortex, right temporal lobe, and right cerebellar hemisphere (Figure 1). Enhanced MRI of the head on January 7, 2024 showed abnormal signals in the lateral temporal lobe and the right cerebellar hemisphere, demyelinating lesions were considered, and encephalitis was considered (Figure 2). On January 30, 2024, the reexamination of head MRI + MRA showed “abnormal signals in the brain stem, right cerebellar hemisphere and bilateral temporal lobes, possible encephalitis, and demyelinating lesions to be excluded.” A diagnosis of demyelinating encephalopathy was made and no special treatment was given. Later, the patient developed difficulty in speech expression and progressive dysphagia. For further diagnosis and treatment, the patient went to our hospital on February 3, 2024. After admission, the patient’s high-sensitivity C-reactive protein: 212.61 mg/L, white blood cell count: 14.9 × 10^9/L, neutrophil percentage: 80.8%, procalcitonin: 21.45 ng/mL, interleukin 6: 159.09 pg/mL. Considering that the severity of the infection, with the high possibility of bacterial infection, might not allow for a fuller etiologic diagnosis because of the urgency of treatment, we selected empirical anti-infective meropenem. The patient was treated with 0.5 g q12h ivgtt meropenem for anti-infection, and other symptomatic support such as eliminating phlegm and relieving cough, acid-reducing and gastric protection, and daily peritoneal dialysis.

**FIGURE 3:**
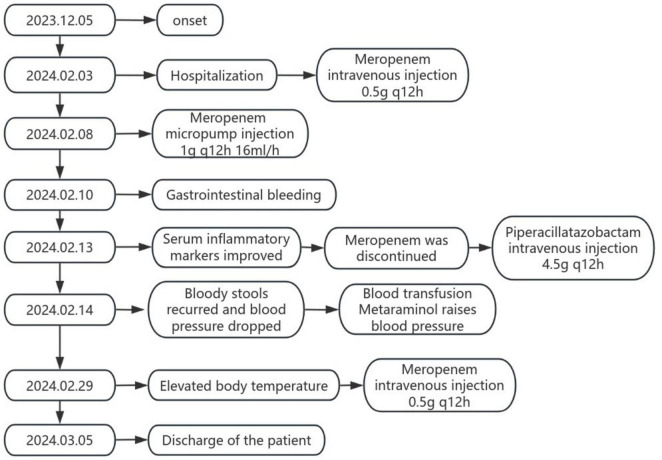
Flow chart.

On February 5, 2024, after excluding relevant contraindications, the patient underwent lumbar puncture under local anesthesia, which showed colorless and clear cerebrospinal fluid outflow, and the cerebrospinal fluid pressure(CSF) was 80 mmH2O. The initial CSF report revealed a WBC count of 1 cell per μL, lactate dehydrogenase of 2.05 mmol per liter, protein of 555 mg per liter, chloride of 121 mmol per liter, and glucose of 4.45 mmol per liter). No Cryptococcus neoformans and acid-fast bacilli were found in cerebrospinal fluid. Cerebrospinal fluid was negative for paraneoplastic antibodies, autoimmune encephalitis antibodies, and demyelinating antibodies. Cerebrospinal fluid metagene testing suggested *Ralstonia mannitolilytica* with a sequence number of 463 and an estimated pathogen concentration of <10^3^(copies/ml) (Table 1). Meanwhile, metagene testing in the blood revealed Escherichia coli with a sequence number of 53 and an estimated pathogen concentration of less than 10^3^(copies/ml) (Table 1). The patient was considered to have intracranial infection, and on February 8, 2024, meropenem was given an increased dose of 1 g q12h 16 ml/h micro-pump injection for anti-infection treatment. On February 10, 2024, the patient had bloody stool with bright color and blood clots at night. The anorectal surgeon and gastroenterologist were requested to assist in the diagnosis and treatment. Colonoscopy and mesenteric artery computed tomography angiography(CTA) were recommended, and gastroscopy was performed simultaneously when necessary. After full consultation with the patient’s family members, colonoscopy was performed, and it was found that the rectal ulcer was bleeding, so the bleeding was stopped by electrocoagulation under the endoscope. On February 13, 2024, the patient’s re-test blood test showed high-sensitivity C-reactive protein: 22.64 mg/L, white blood cell count: 7.6 × 10^9/L, and neutrophil percentage: 85.1%. Considering that the patient’s infection gradually improved under the treatment of meropenem, the inflammatory indicators in the blood tests were significantly lower than before, and the temperature did not rise again, the antibiotic was adjusted to piperacillin-tazobactam 4.5 g q12h ivgtt. On February 14, 2024, the patient developed bloody stool again with decreased blood pressure. After consultation, his family refused to undergo colonoscopy and interventional surgery again, and continued to receive conservative treatment, blood transfusion and metanolamine vasopressor treatment. Later, the patient showed a progressive decrease in hemoglobin due to active gastrointestinal bleeding. The patient’s family members showed understanding and refused to be transferred to the intensive care unit for further diagnosis and treatment. On February 29, 2024, the patient’s body temperature increased again, and the serum infection indicators were higher than before (hypersensitive C-reactive protein: 119.23 mg/L, white blood cell count: 9.9 × 10^9/L, neutrophil percentage: 89.5%, procalcitonin: 9.21 ng/mL), piperacillin tazobactam was stopped, and the patient’s renal insufficiency was considered. Meropenem 0.5 g q12h ivgtt was given again for anti-infection. On March 05, 2024, the patient was in critical condition, and the patient’s condition was not significantly relieved after active treatment, so the family decided to discharge. We simplified the entire course of the patient’s illness and treatment into a flow chart (Figure 3).

**TABLE 1:** The results of mNGS.

Type of specimen	Types of bacteria	Name of pathogen	Number of sequences	Estimated pathogen concentration (copies/ml)
Cerebrospinal fluid	G-	*Ralstonia mannitolilytica*	463	<10^3^
Serum	G-	*Escherichia coli*	53	<10^3^

## Discussion

*Ralstonia mannitolilytica* is an aerobic Gram-negative non-fermentative bacterium commonly found in water and soil, which has become a conditional pathogen causing infection in immunocompromised patients, hemodialysis patients or patients with respiratory problems, patients with indwelling equipment, and newborns (7, 9). *Ralstonia mannitolilytica* was first reported in 1995, and since then there have been case reports of infection involving newborns, tumors, dialysis and kidney transplant patients (8, 10–14). Most of these are associated with the use of contaminated solutions, including saline, water for injection, and disinfectants (7, 15). Common sources of contamination in hospitals are dialysis water, sterile water for injection, and oxygen supply systems (8, 11, 13).

There is only one reported case of meningitis caused by *Ralstonia mannitolilytica* (4), which occurred after ventricular drainage. Our case presented with symptoms of cerebellar and throat muscle dysfunction as the first manifestation. With the gradual progress of the disease, physical activity disorder occurred. Since this patient had no obvious history of infection before the onset of the disease and presented with neurological dysfunction as the first manifestation, serologic examination did not reveal signs of infection, and the findings of brain MRI were more likely to be demyelinating lesions. Based on the clinical features, imaging findings and laboratory tests of *Ralstonia mannitolilytica*, the patient was almost misdiagnosed as cerebral demyelinating disease because it was atypical compared with the previous reported cases of *Ralstonia mannitolilytica* with special imaging manifestations and multiple complications. Our patient had a number of triggers that triggered the infection with *Ralstonia mannitolilytica*. First, this patient had uremia and was undergoing long-term intermittent peritoneal dialysis. Although previous outbreaks have involved dialysis machines, this is an isolated case.

At present, the diagnosis of meningoencephalitis caused by *Ralstonia mannitolilytica* infection mainly relies on metagene detection. Traditionally, the diagnosis of infectious diseases has often had limitations (16), including time consumption, lack of specificity, and the unculturability of some organisms. Metagenomics is more advantageous than traditional diagnostic methods for infectious diseases (17). Clinical mNGS is revolutionating diagnostic techniques, particularly in intensive care units, where rapid and precise pathogen identification is essential (18). It can directly sequence the base sequence in the sample, with rapid and high accuracy, and is less affected by the use of antibiotics and the body’s immune status (19). From a diagnostic perspective, identification and removal of bacterial, fungal, and viral genomic contamination are essential for NGS sequencing. With the development of NGS technology and the proposal of solutions to solve the limitations of sequencing, clinicians are recommended to test specimens as early as possible when encountering unexplained infection, suspected infection of vital organs, or rapid disease progression (20). However, the lack of this detection technology in primary hospitals may delay the diagnosis of some patients with intracranial infection.

Despite its low virulence, the resistance to antibiotics is high, in part due to its ability to form biofilms, and laboratory identification of it is sparse, making it a difficult organism to treat (2). Currently, there are no clear guidelines for the treatment of *Ralstonia mannitolilytica* infection. Treatment regimens need to be carefully planned because the genus produces various enzymes that can hydrolyze antibiotics, and resistance to aminoglycosides and lactams is frequently reported (2). Particular attention has been paid to the rise in resistance to many modern antibiotics such as ceftazidime, attreonam, and carbapenems (2, 21). Because of the presence of uremia, the patient was treated with meropenem with continued peritoneal dialysis. According to the feedback of the patient’s symptoms and serum inflammatory indicators, it can be observed that the meropenem 1 g q12h 16 ml/h micro-pump injection anti-infective treatment program has a great improvement in the patient’s condition. Later, due to the patient’s long-term bed rest and immunodeficiency, serious complications occurred, which led to multiple organ failure.

Through the signs and symptoms, related imaging characteristics and complications, it is still necessary to combine a large number of clinical data to distinguish it from other similar diseases and obtain more appropriate treatment and critical care plans. We hope that this case will attract more attention and provide relevant reference for the diagnosis and treatment of other similar patients.

## Data Availability

The original contributions presented in this study are included in this article/supplementary material, further inquiries can be directed to the corresponding authors.
